# An autopsied case of MM1-type sporadic Creutzfeldt-Jakob disease with pathology of Wernicke encephalopathy

**DOI:** 10.1080/19336896.2018.1545525

**Published:** 2018-11-14

**Authors:** Yasushi Iwasaki, Rina Hashimoto, Yufuko Saito, Ikuko Aiba, Akira Inukai, Akio Akagi, Maya Mimuro, Hiroaki Miyahara, Tetsuyuki Kitamoto, Mari Yoshida

**Affiliations:** a Department of Neuropathology, Institute for Medical Science of Aging, Aichi Medical University, Nagakute, Japan; b Department of Neurology, National Hospital Organization Higashinagoya National Hospital, Nagoya, Japan; c Division of CJD Science and Technology, Department of Neurological Science, Tohoku University Graduate School of Medicine, Sendai, Japan

**Keywords:** Creutzfeldt-Jakob disease, mammillary body, spongiform change, thiamine deficiency, Wernicke encephalopathy

## Abstract

An 83-year-old Japanese man presented with gait disturbance followed by rapidly-progressive cognitive impairment. Magnetic resonance diffusion-weighted images showed extensive hyperintense regions in the cerebral cortex. Four weeks after symptom onset, myoclonus appeared, and the patient developed difficulty swallowing; intravenous peripheral continuous infusions without vitamin supplementation were administered during the last two months of the patient’s life. The patient reached the akinetic mutism state and died 12 weeks after symptom onset due to sepsis. The brain weighed 940 g and showed general cerebral atrophy. Extensive spongiform change were observed in the cerebral cortex, striatum, thalamus, and cerebellar cortex, but gliosis was generally mild. Numerous newly-developed hemorrhage foci were observed in the mammillary body, the areas adjacent to the third and fourth ventricles, and the periaqueduct of the midbrain; however, proliferation of capillaries and endothelium and collections of macrophages were relatively inconspicuous. These findings suggested comorbidity with the acute stage of Wernicke encephalopathy (WE). Immunostaining showed extensive diffuse synaptic-type prion protein deposition in the gray matter. According to the neuropathological, genetic, and molecular findings, the present case was finally diagnosed as MM1-type sporadic Creutzfeldt-Jakob disease (CJD) with WE. We should remain alert to the diagnosis of WE when CJD is suspected, and it is necessary to consider the complications of both diseases. This report emphasizes the importance of pathological investigations for the diagnosis of CJD, WE, and the coexistence of both.

## Introduction

Typical Creutzfeldt-Jakob disease (CJD) cases show a rapidly-progressive clinical course with cognitive dysfunction, myoclonus, periodic sharp-wave complexes (PSWCs) on electroencephalography (EEG), and hyperintensity in the cerebral cortex and striatum on magnetic resonance diffusion-weighted images (MR-DWI) in the early disease stage, and patients reach the akinetic mutism state within several months after symptom onset [1–4]. In cases where these characteristic clinical findings are recognized, the probable diagnosis of CJD is relatively easy [1–3]; however, neuropathological investigation is absolutely needed for definitive diagnosis [1,3,5].

We experienced an autopsy case of MM1-type sporadic CJD with pathology of Wernicke encephalopathy (WE). It is generally considered that WE is less frequent in Japan than in North American and European countries [6,7]. Furthermore, to the best of our knowledge, although several CJD with WE cases have been reported in North American and European countries, no similar case has been reported in Japan. Here, we report the detailed clinicopathological findings of such a case with a literature review, particularly regarding the association between CJD and WE.

## Clinical summary

**Figure 1.**
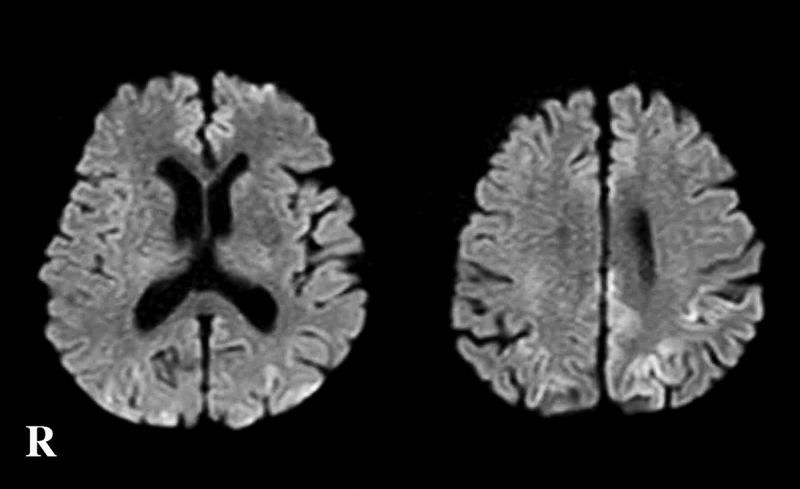
Magnetic resonance diffusion weighted images obtained 2 weeks after the onset of symptoms. Extensive hyperintense regions are apparent in the bilateral cerebral cortex. Findings pointing toward Wernicke encephalopathy are not recognized.R, right side.

An 83-year-old healthy Japanese man presented with gait disturbance, and cognitive impairment appeared the following week. Two weeks after symptom onset, he was admitted to the Department of Neurology due to rapidly-progressing cognitive dysfunction. His past medical history was unremarkable; there was no history of surgical operations, alcoholism, or malnutrition. He also had no family history of prion disease or iatrogenic exposure to CJD. The initial neurological examination revealed unstable gait, but eye-movement restrictions, nystagmus, muscle weakness, or sensory disturbance were not recognized. He was confused with poor general knowledge and impaired short, immediate, and long-term memories. MR-DWI showed extensive hyperintense regions in the cerebral cortex (Figure 1). Routine laboratory serum tests were normal. His neurological symptoms deteriorated rapidly and was eventually unable to communicate. Four weeks after symptom onset, myoclonus appeared, and he developed difficulty swallowing. A clinical diagnosis of probable CJD was made according to the diagnostic criteria [3]. The patient’s family hoped not to enforce tube-feeding or intravenous hyperalimentation and opted for peripheral continuous infusions without vitamin supplementation as a symptomatic treatment. Intravenous-infusion feeding was provided during the last two months of the patient’s life; a total of 1500 ml hydration fluids of hypotonic crystalloid solution were infused every day until death. The patient reached the akinetic mutism state 7 weeks after symptom onset, and the myoclonus became severe in degree at this stage. The patient developed aspiration pneumonia 10 weeks after symptom onset and antibiotic instillation was performed, but the patient died due to sepsis after 12 weeks of total disease duration. The serum level of thiamine (vitamin B1) was not assessed. Because of consciousness disturbance, the evaluation of eye-movement disorders was difficult in the later disease stage.

## Methods

### Neuropathological examination

An autopsy was performed, and the brain was fixed in 20% formalin for four weeks. After sectioning and trimming, the brain tissues were immersed in 95% formic acid for 1 hour to inactivate prion infectivity. Then, the specimens were embedded in paraffin and cut into 4-μm sections. The sections were deparaffinized in lemosol, rehydrated through an ethanol gradient, and stained. For routine neuropathological examinations, the sections were processed with hematoxylin-and-eosin, Klüver-Barrera, and modified Gallyas-Braak (G-B) silver stainings.

Immunohistochemical analysis of selected sections was carried out with anti-PrP antibody (3F4; Dako, Glostrup, Denmark, mouse monoclonal, diluted 1:100) after hydrolytic autoclaving for antigen retrieval. The PrP immunostaining protocol using the EnVision amplified method (EnVision plus kit, Dako) was conducted as previously described [8]. Immunostaining with anti-Aβ (4G8; Signet, Dedham, MA, mouse monoclonal, diluted 1:2,000), anti-hyperphosphorylated tau (AT-8; Innogenetics, Ghent, Belgium, mouse monoclonal, diluted 1:1,000), and anti-phosphorylated α-synuclein (pSyn#64; Wako Pure Chemical Industries, Osaka, Japan, mouse monoclonal, 1:3,000) was also performed. Peroxidase-conjugated streptavidin binding was visualized using 3,3ʹ-diaminobenzidine (Wako Pure Chemical Industries) as the final chromogen. Immunostained sections were counterstained with Mayer’s hematoxylin.

### Western blot analysis of protease-resistant PrP and PrP gene analysis

The cryopreserved right frontal cerebral cortex, which was snap frozen and stored at −80°C prior to use, was homogenized, and western blot analysis of protease-resistant PrP (PrP^Sc^) was performed using 3F4 antibodies as previously described [8]. PrPSc typing was performed according to the sporadic CJD classification system proposed by Parchi et al. [9]. Furthermore, to investigate the co-occurrence of type 1 and type 2 PrPSc, we carried out an additional experiment using the type 1 PrPSc-specific antibody Tohoku-1 and the type 2 PrPSc-specific antibody Tohoku-2 after protease treatment [10].

Genomic DNA was extracted from the cryopreserved brain tissue and used to amplify the open reading frame of the PrP gene using polymerase chain reaction. We searched for mutations and investigated the polymorphisms at codons 129 and 219 using restriction fragment length polymorphism analysis, as previously described [8]. The patient’s DNA was also used for apolipoprotein E genotyping.

### Retrospective research regarding prion disease with WE

More than 5000 cases were neuropathologically analyzed during the period between 1976 and 2017 at the Institute for Medical Science of Aging, Aichi Medical University. Among these serial autopsy cases, we searched for those neuropathologically-diagnosed as cases of CJD with WE. The neuropathological examinations had been performed previously and were not a part of the present study.

## Results

### Macroscopic findings

**Figure 2.**
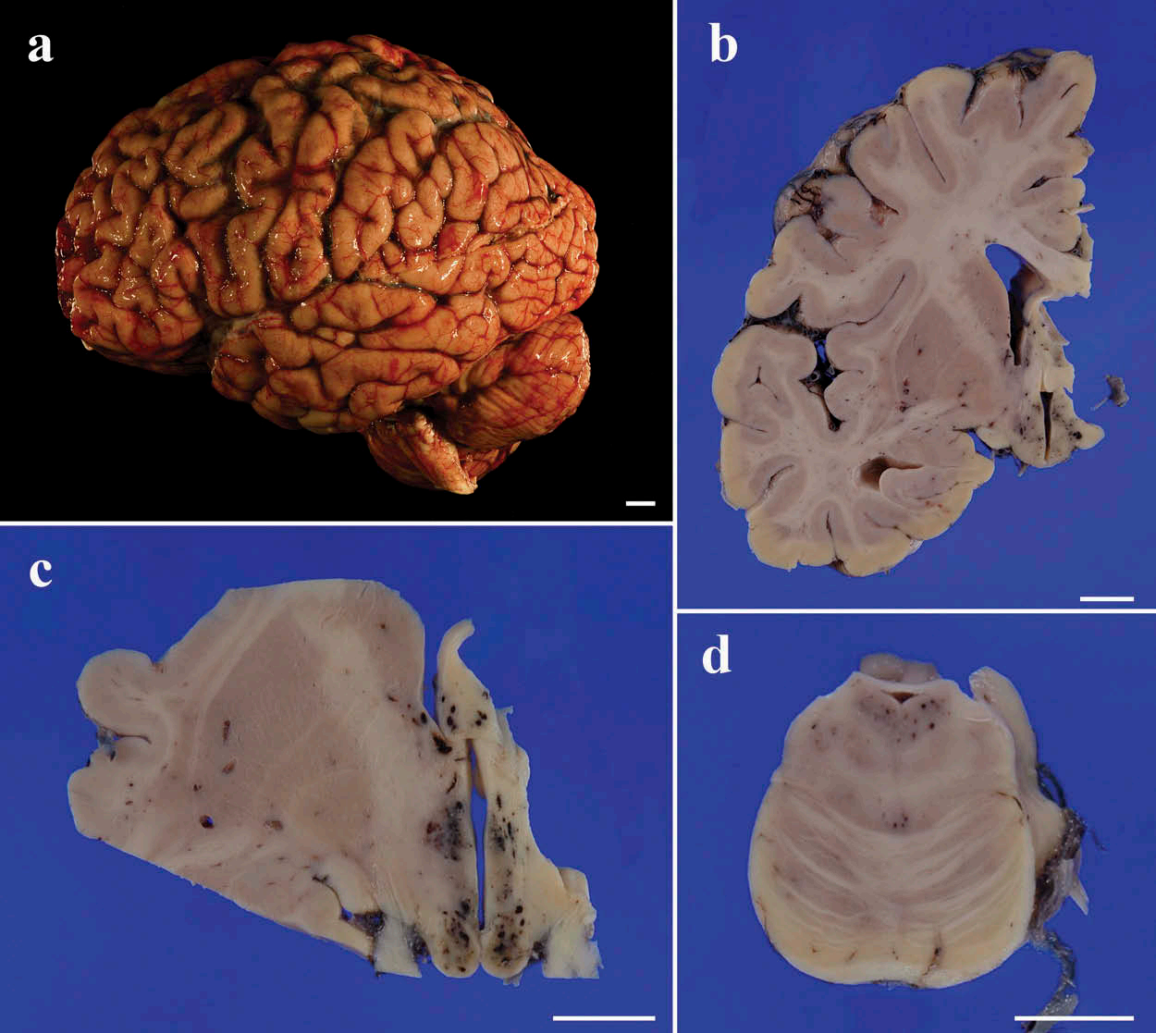
Macroscopic appearance of the brain. (a). Before fixation, the cerebrum shows general moderate atrophy with widening of the sulci, particularly in the frontal lobe. The cerebellum also shows general atrophy. (b). A coronal section of the left cerebral hemisphere shows mild thinning of the neocortex with mild dilatation of the lateral ventricles. The striatum, white matter, and hippocampus are relatively preserved from the atrophy. Numerous small petechiae are recognized in the hypothalamus. (c). A low-power field image of the coronal section at the level of the mamillary body shows numerous petechiae in the mamillary body, hypothalamus, and column of the fornix (d). Low-power field image of the horizontal section of the pons also shows several petechiae in the tegmentum. Scale bars = 10 mm.

The brain weighed 940 g before fixation. Symmetrical moderate atrophy of the cerebrum was observed (Figure 2(a)). Coronal sections of the cerebral hemisphere revealed mild thinning of the neocortex and mild dilatation of the lateral ventricles system. Numerous punctuate petechial hemorrhages in the mammillary body and areas adjacent to the third ventricle were noted (Figure 2(b,c)). The cerebellum showed general atrophy, whereas the brainstem was preserved from atrophy. Horizontal sections of the brainstem revealed similar petechial hemorrhages in the periaqueductal area of the midbrain and around the fourth ventricle in the pons and medulla (Figure 2(d)). Depigmentation was not apparent in the substantia nigra or in the locus coeruleus. Pseudohypertrophy of the inferior olivary nucleus was not recognized.

### Microscopic findings

**Figure 3.**
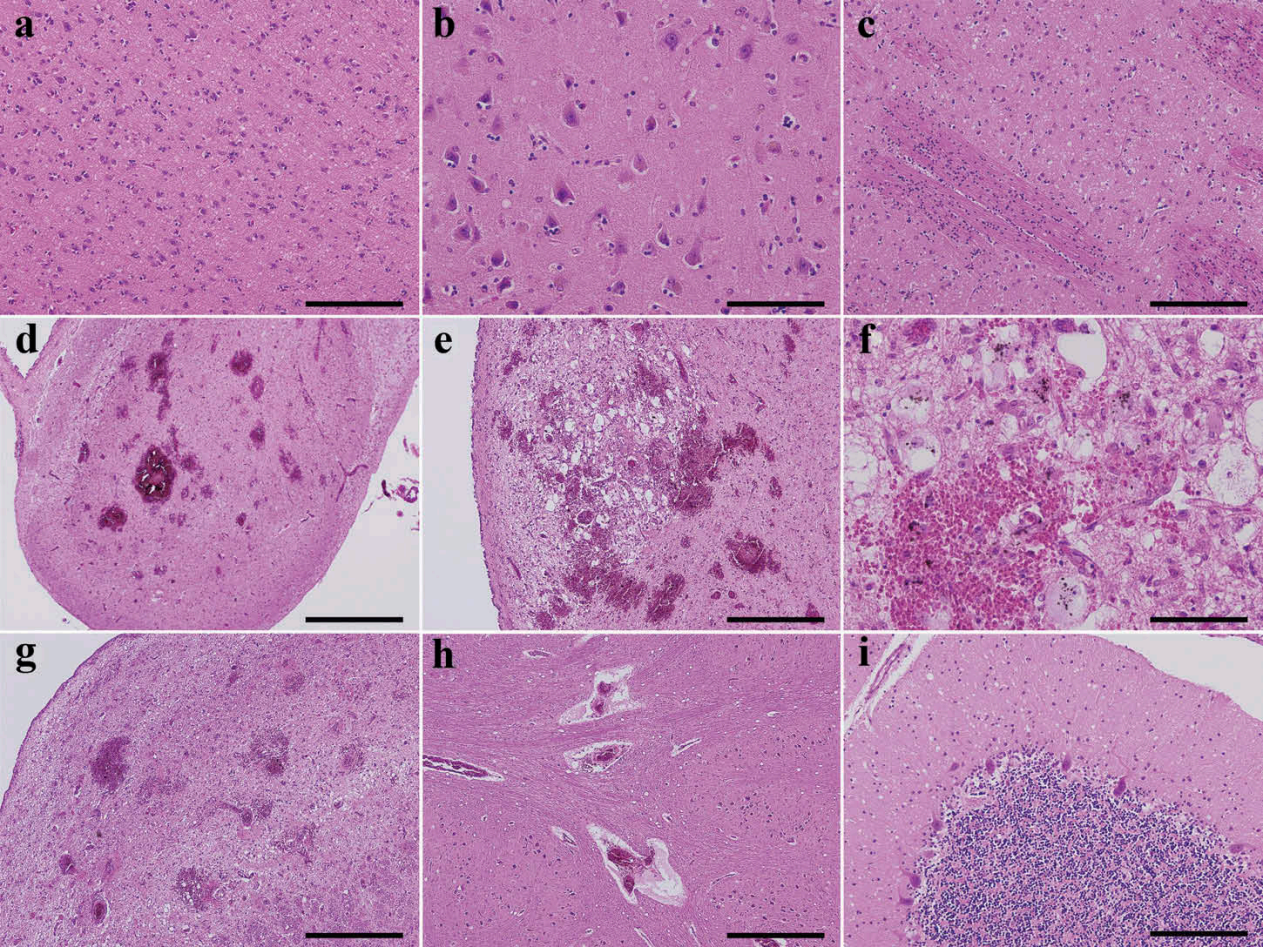
Representative microscopic findings by hematoxylin-and-eosin staining. (a). The cerebral neocortex shows numerous small round vacuoles with a clear boundary in the neuropil. Gliosis and hypertrophic astrocytosis are generally mild (superior temporal gyrus). (b). The subiculum shows mild spongiform change without apparent gliosis. (c). The putamen shows apparent spongiform change and gliosis with hypertrophic astrocytosis. The pencil fibers are largely preserved. (d). The mammillary body shows numerous newly-developed hemorrhage foci. (e). The areas adjacent to the third ventricle of the hypothalamus also shows numerous newly-developed hemorrhage foci with vacuolation. The size and morphology of the vacuoles do not resemble the spongiform change seen in CJD. (f). The proliferation of capillaries and endothelium and collections of macrophages containing iron pigment are relatively inconspicuous around the hemorrhage foci (hypothalamus). (g). In the tegmental region of the pons (fossa rhomboidea), many small hemorrhages are recognized. (h). The inferior olivary nucleus is relatively preserved from neuronal loss, but perivascular microhemorrhages are observed in adjacent areas. (i). Extensive spongiform change are observed in the molecular layer of the cerebellum. The Purkinje cell layer is preserved, whereas the granule cell layer shows moderate neuronal loss. Scale bars; a, b, i, e: 200 μm, c, f: 200 μm, d: 1 mm, e, g, h: 500 μm.

In the cerebral neocortex, fine vacuole-type spongiform change was extensively observed (Figure 3(a)). Gliosis and hypertrophic astrocytosis were also extensively observed, but the degree was generally mild. Tissue rarefaction of the neuropil and neuronal loss were not so apparent. Florid or Kuru plaques were not observed. The hippocampal formation and subiculum showed mild spongiform change without apparent gliosis (Figure 3(b)). The striatum showed apparent spongiform change and gliosis (Figure 3(c)), whereas the globus pallidus and the lateral thalamus were relatively preserved. The mammillary body, medial thalamus, and hypothalamus showed numerous newly-developed hemorrhage foci (Figure 3(d)). The areas around the hemorrhage foci showed vacuolation, but the size and morphology did not resemble spongiform change of the cerebral cortex (Figure 3(e)). Proliferation of capillaries and endothelium and collections of macrophages containing iron pigment were relatively inconspicuous (Figure 3(f)). Myelin pallor or gliosis was not so apparent in the cerebral white matter. In the brainstem, gliosis and neuronal loss were not apparent in the substantia nigra, pontine nucleus, locus coeruleus, and dorsal nucleus of the vagus. In the periaqueductal area and in the tegmental region, many small hemorrhages, which extended to below the fourth ventricle in the medulla, were recognized (Figure 3(g)). The inferior olivary nucleus was relatively preserved, but perivascular small hemorrhages were observed in adjacent areas (Figure 3(h)). Pyramidal tract degeneration was not observed. In the cerebellum, the molecular layer showed extensive spongiform change (Figure 3(i)). The granule cell layer showed moderate neuronal loss, while the Purkinje cell layer was preserved. The dentate nucleus showed gliosis, but neurons were preserved in number. The cerebellar white matter was preserved.

### PrP immunohistochemical findings

**Figure 4.**
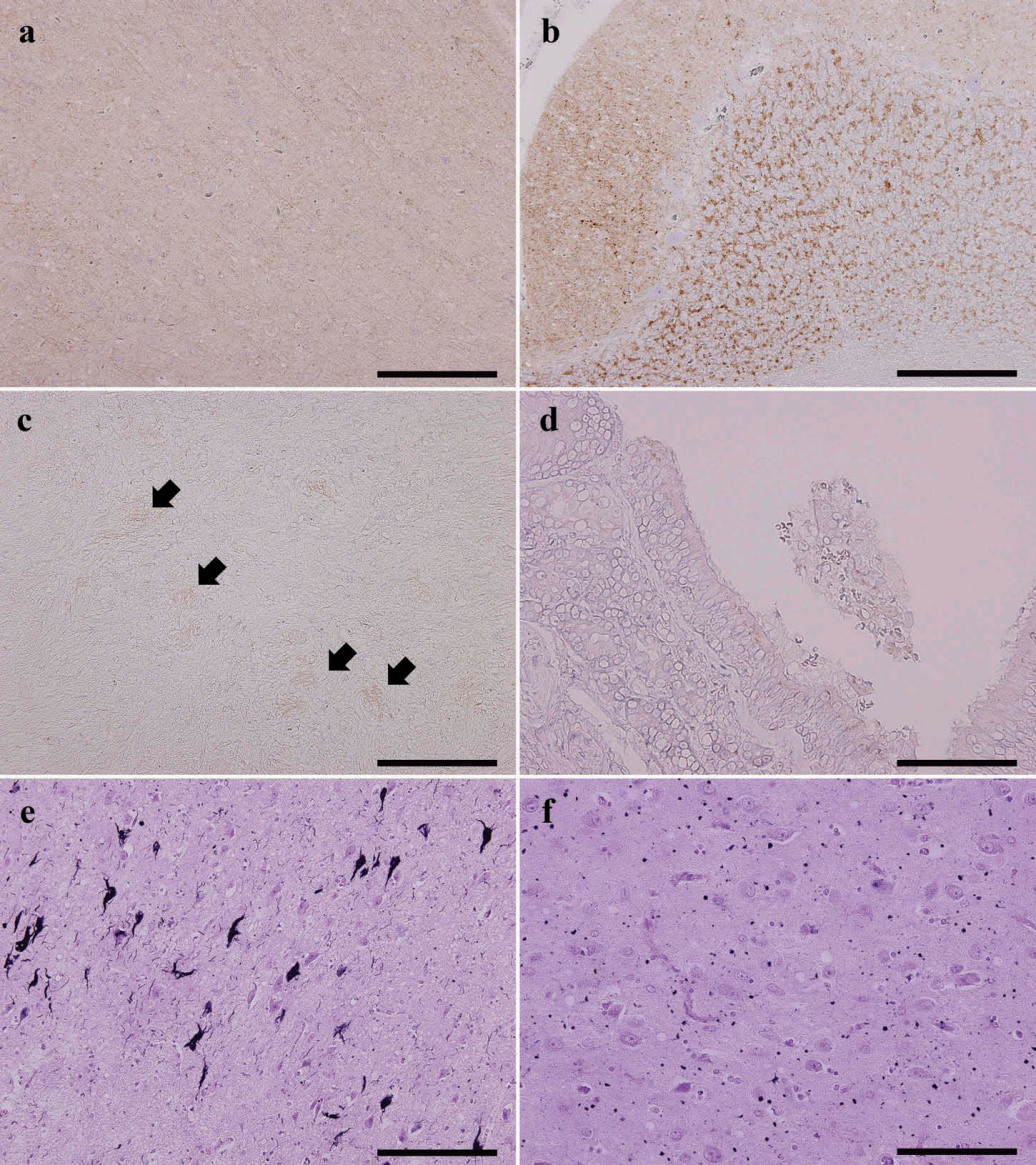
Representative microscopic findings by PrP immunostaining and G-B silver staining. (a,b). PrP immunostaining shows a diffuse fine granular staining pattern (synaptic-type) (A; superior temporal gyrus, B; cerebellar cortex). (c,d). PrP deposition is also observable in the olfactory bulb, particularly in the glomerulus (arrows) (C) and the olfactory mucosa (D). (e). The transentorhinal cortex in the parahippocampal gyrus shows several neurofibrillary tangles with neuropil threads. (f). Many argyrophilic grains are identified in the amygdala. A–D; anti-PrP (3F4) immunostaining, E, F; Gallyas-Braak (G-B) silver staining,Scale bars; A–C, E: 200 μm, D, F: 100 μm.

Immunostaining for PrP revealed diffuse synaptic-type PrP deposition in the cerebral cortex (Figure 4(a)), basal ganglia, thalamus, substantia nigra, pontine nucleus, inferior olivary nucleus, and cerebellar cortex (Figure 4(b)). PrP deposition was also recognized in the olfactory bulb (Figure 4(c)) and olfactory mucosa (Figure 4(d)).

### Aging pathology

A few neurofibrillary tangles were observed using G-B staining and AT-8 immunostaining in the limbic system and the nucleus basalis of Meynert, and the distribution corresponded to Braak stage II (Figure 4(c)) [11,12]. Senile plaques were hardly identified using G-B staining and anti-amyloid β immunostaining. Amyloid angiopathy was not recognized. By using G-B staining, argyrophilic grains were mildly observed in the amygdala, hippocampal formation, and parahippocampal gyrus, and the distribution corresponded to Saito stage I (Figure 4(d)) [13]. Lewy bodies and α-synuclein pathology were absent as revealed by anti-α-synuclein immunostaining. No apparent vascular lesions were recognized in the brain.

### Western blot analysis of protease resistant PrP and PrP gene analysis

**Figure 5.**
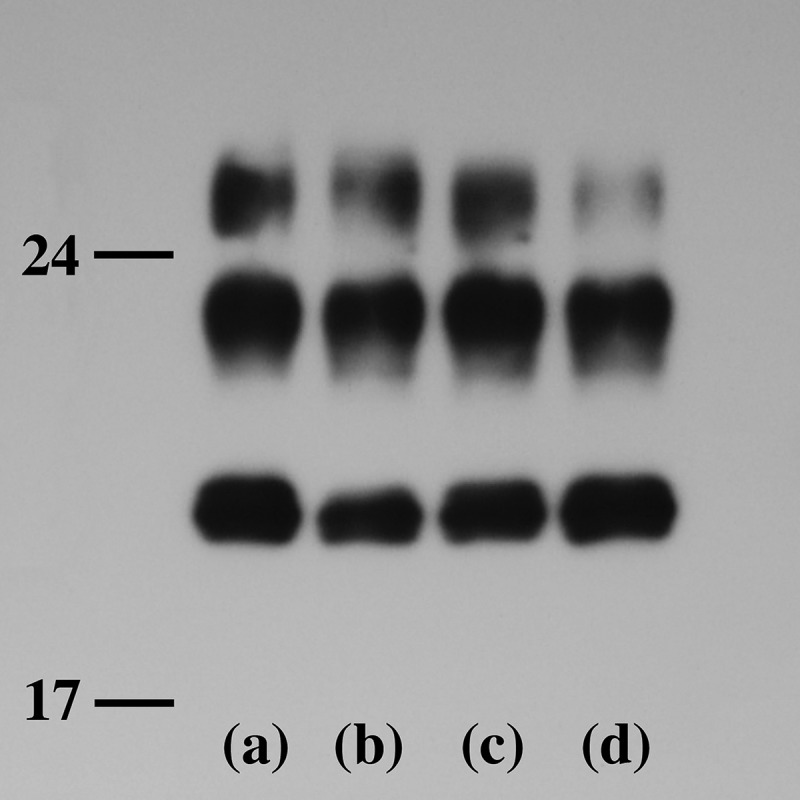
Western blot analysis of proteinase K-resistant PrP. The gel mobility of PrP^Sc^ of the present patient (lane a) is compared with that from three patients with MM1-type sCJD (lanes b, c, and d). In all lanes, PrP^Sc^ migrated as three bands identified as the diglycosylated (upper band), monoglycosylated (middle band), and unglycosylated forms (lower band). The nonglycoform bands show type 1 PrP^Sc^ located at 21 kilodaltons.

Western blot analysis indicated the presence of type 1 PrP^Sc^, with a relative molecular mass of the unglycosylated band of approximately 21 kDa (Figure 5). Western blot analyzes using type 1 PrP^Sc^-specific antibodies showed apparent positive bands, whereas those using type 2 PrP^Sc^-specific antibodies showed no positive bands (data not shown).

No mutations in the PrP gene open reading frame were identified. It was determined that methionine at polymorphic codon 129 was homozygous. Polymorphic codon 219 was homozygous for glutamic acid. Apolipoprotein E gene analysis revealed epsilon 3 homozygosity.

### Retrospective study regarding prion disease with WE

One hundred and forty-four pathologically-confirmed prion-disease cases with adequate neuropathological investigation of the brain were located in the autopsy record of our institute (including the present case): 119 sporadic CJD cases (including 25 cases without PrP genetic analysis), 11 genetic CJD cases (seven V180I genetic CJD cases and four M232R genetic CJD cases), eight dura mater graft-associated CJD cases, and six Gerstmann-Sträussler-Scheinker disease cases (two cases showed P102L PrP gene mutation and four cases without PrP gene analysis). Biopsy- and necropsy-diagnosed cases were excluded. Cases with insufficient cerebral pathological investigations were also excluded. The only identified case with prion disease with WE was the one described here.

## Discussion

In the present case, we were surprised by the pathological finding of WE, a treatable disease related to thiamine deficiency, during the pathological examination of a CJD case. Because WE was not clinically suspected before death, the thiamine level in the serum was not measured. We only noticed the WE on autopsy. In general, pathological diagnosis of WE is established based on prominent small blood vessels with endothelial proliferation, perivascular petechial hemorrhages, and reactive gliosis with relative preservation of neurons visible in the mamillary body, areas adjacent to the third and fourth ventricles, and the periaqueduct [14,15]. In the present case, numerous newly-developed hemorrhage foci were observed in these regions; however, proliferation of capillaries and endothelium and collections of macrophages were relatively inconspicuous. These findings suggested that the pathology corresponded to the acute stage of WE and probably emerged at the CJD terminal stage. In addition to the neuropathological findings, and based on the genetic and molecular findings, the present case was finally diagnosed as MM1-type sporadic CJD with WE.

It has been suggested that there is difficulty in clinically discriminating WE from CJD [16]. Furthermore, the comorbidity of both disorders is not an exceptional occurrence [16,17]. In 1988, Gaytan-Garcia et al. reported the clinicopathological findings of three CJD cases with WE [17]. Bertrand et al. investigated the prevalence of WE in 657 autopsied CJD suspected cases referred from 2001 to 2006 to the French Neuropathology Network of CJD: 434 were diagnosed as CJD and 223 were not [16]. In the study, WE was combined with CJD in 14 cases, including 13 sporadic and one iatrogenic cases. Interestingly, in five (0.76% of suspected CJD cases and 2.24% of pathologically-confirmed non-CJD cases), the final pathological diagnosis was isolated WE without CJD pathology. In WE associated with sporadic CJD, the CJD disease duration was relatively longer (4–24 months, median: 14 months), and they speculated that the prolonged disease duration explained the occurrence of thiamine deficiency. In their observations, positive findings of proteinase-K resistant PrP could be obtained by western blot analysis in 12 CJD with WE cases, of which 11 showed type 2 PrP and one showed type 1 + 2. Although, no patient with MM1-type sporadic CJD was found in their study, the present case was of the MM1-type; the most frequent and that which has the shortest disease duration [9]. The clinicopathological presentation of sporadic CJD is influenced by the polymorphisms of PrP gene codon 129 (methionine [M] or valine [V]) and prion strain (type 1 PrP^Sc^ or type 2 PrPSc) [1,8,9]. A classification of sporadic CJD into six distinct subtypes (MM1, MM2, MV1, MV2, VV1, and VV2) has been proposed, and the MM1 type comprises the most common clinical and pathological findings [9]. Regarding our institution, the prevalence of prion disease with WE was 0.69% (1/144) and that of CJD with WE was 0.72% (1/138). This prevalence is lower than that reported by the French study [16].

Thiamine deficiency can occur rapidly if body stores are depleted and it results in WE [14,15,18]. WE is commonly associated with chronic alcoholism, but has also been reported in an array of conditions that affect nutrition including prolonged intravenous feeding, malignancies, infections, acquired immunodeficiency syndrome, starvation, hyperemesis gravidarum, and long-state dementia [14,15,18,19]. Among the undiagnosed WE cases, the high percentage of non-alcoholic patients stresses the necessity of remaining alert to this diagnosis [19], especially when a noncurable disease is suspected.

We previously reported on the clinicopathologic investigations of autopsy-confirmed WE cases in our institution (we had no experience with the present case at the point) [6]. In that study, we found 19 WE cases (0.41%) from the total 4,630 brain-autopsy files. The frequency was lower than that reported by a similar study based on European and American autopsy records [15,18]. Background disorders included seven cases of neurological disorders (two with Huntington’s disease and one each for multiple system atrophy, dementia with Lewy bodies, progressive supranuclear palsy, familial amyloid polyneuropathy, and anterior spinal artery syndrome), five cases of chronic alcoholism, five cases of malignancies, one case of hyperemesis gravidarum, and one case of sepsis [6]. Only five patients were clinically diagnosed with WE prior to death according to the clinical findings and/or MRI findings [6].

Two CJD cases clinically mimicking WE have been reported [20,21]. The clinical symptoms and signs of WE and CJD partly overlap; ophthalmoplegia, ataxia, and consciousness disturbances are the main findings in WE [22], whereas rapidly-progressive dementia associated with neurological disturbances including ataxia raises the suspicion of CJD [1,2]. Eye-movement disorders including ophthalmoplegia are sometimes observed in definite CJD cases [23]. Although the present case showed gait unsteadiness at disease onset, we considered that the symptom was induced by CJD, not WE because no apparent findings pointing toward WE, such as MRI findings and ophthalmoplegia, were not recognized at that time.

CJD directly contributed to the nutritional deficiency due to several factors, such as consciousness disturbance, cognitive impairment, dysphagia, and the later akinetic mutism state. Because the present patient was of advanced age, the thiamine stores may have been somewhat depleted. The interruption of oral feeding and use of parenteral dextrose as the sole caloric supply during the akinetic mutism state would profoundly affect thiamine stores. Furthermore, we considered the possibility of excessive thiamine depletion due to the severe myoclonus and sepsis. Intravenous carbohydrate loading and individual susceptibility to thiamine deficiency may have also influenced the development of WE.

In the present case, PrP deposition could be observed in the olfactory bulb and olfactory mucosa by immunostaining. The olfactory bulb is connected to the olfactory mucosa through the olfactory nerve and the latter is in contact with the external environment. One report showed that PrPSc could be detected in the olfactory mucosa [24]. We wonder whether the olfactory bulb may be a possible route of PrP infection from the olfactory mucosa not only in the present case but also in some sporadic CJD cases.

WE-type pathology may be more common than suspected in patients with CJD and other disorders accompanied by dementia. In these disorders, WE may develop surreptitiously without clinically-obvious episodes or perhaps its expression may be masked by symptoms of cognitive dysfunction or consciousness disturbance. We should remain alert to the diagnosis of WE when CJD is suspected, and CJD should be considered in patients with WE. Moreover, it is necessary to consider the complications of both diseases; in cases with both diseases, a pathological investigation is absolutely needed. This report emphasizes the importance of pathological investigations for the diagnosis of CJD, WE, and the coexistence of both.
